# Effect of park prescriptions with and without group visits to parks on stress reduction in low-income parents: SHINE randomized trial

**DOI:** 10.1371/journal.pone.0192921

**Published:** 2018-02-15

**Authors:** Nooshin Razani, Saam Morshed, Michael A. Kohn, Nancy M. Wells, Doug Thompson, Maoya Alqassari, Amaka Agodi, George W. Rutherford

**Affiliations:** 1 Center for Nature and Health, UCSF Benioff Children’s Hospital Oakland, Oakland, California, United States of America; 2 Departments of Epidemiology and Biostatistics and Orthopedic Surgery, School of Medicine, University of California at San Francisco, San Francisco, United States of America; 3 Department of Design & Environmental Analysis, College of Human Ecology, Cornell University, Ithaca, New York, United States of America; 4 Department of Social Work, University of California at Berkeley, Berkeley, California, United States of America; 5 Division of Primary Care, UCSF Benioff Children’s Hospital Oakland, Oakland, California, United States of America; 6 Department of Epidemiology and Biostatistics, School of Medicine, University of California at San Francisco, San Francisco, United States of America; Karolinska Institutet, SWEDEN

## Abstract

**Introduction:**

Exposure to nature may reduce stress in low-income parents. This prospective randomized trial compares the effect of a physician’s counseling about nature with or without facilitated group outings on stress and other outcomes among low-income parents.

**Materials and methods:**

Parents of patients aged 4–18 years at a clinic serving low-income families were randomized to a supported park prescription versus independent park prescription in a 2:1 ratio. Parents in both groups received physician counseling about nature, maps of local parks, a journal, and pedometer. The supported group received additional phone and text reminders to attend three weekly family nature outings with free transportation, food, and programming. Outcomes measured in parents at baseline, one month and three months post-enrollment included: stress (using the 40-point Perceived Stress Scale [PSS10]); park visits per week (self-report and journaling); loneliness (modified UCLA-Loneliness Scale); physical activity (self-report, journaling, pedometry); physiologic stress (salivary cortisol); and nature affinity (validated scale).

**Results:**

We enrolled 78 parents, 50 in the supported and 28 in the independent group. One-month follow-up was available for 60 (77%) participants and three-month follow up for 65 (83%). Overall stress decreased by 1.71 points (95% CI, -3.15, -0.26). The improvement in stress did not differ significantly by group assignment, although the independent group had more park visits per week (mean difference 1.75; 95% CI [0.46, 3.04], p = 0.0085). In multivariable analysis, each unit increase in park visits per week was associated with a significant and incremental decrease in stress (change in PSS10–0.53; 95% CI [-0.89, -0.16]; p = 0.005) at three months.

**Conclusion:**

While we were unable to demonstrate the additional benefit of group park visits, we observed an overall decrease in parental stress both overall and as a function of numbers of park visits per week. Paradoxically the park prescription without group park visits led to a greater increase in weekly park visits than the group visits. To understand the benefits of this intervention, larger trials are needed.

**Trial registration:**

ClinicalTrials.gov NCT02623855

## Introduction

Chronic, unbuffered stress among low-income parents and children has been recognized as a contributing factor to health outcome disparities [1]. Low-income parents describe intense and daily stressors arising from difficulties in providing for their families’ basic needs such as food and housing; moreover, these are compounded by discrimination, immigration, and mental health issues [2, 3]. Low-income families can be socially isolated and may lack access to community-based buffers for these stressors [4]. With more than 46.7 million people living in poverty in the United States, innovative, community-based means of dealing with stress are needed in this population [5].

The potential health benefit of being exposed to nature for stress relief in low-income communities is a growing field of research [6]. Nature (defined here as “physical features and processes of nonhuman origin, including the ‘living nature’ of flora and fauna, together with still and running water, qualities of air and weather, and the land- scapes” [7]) has been associated with a positive, proportional decrease in stress—as measured by blood pressure, heart rate, cortisol and inflammatory markers [8, 9]. The higher the baseline stress, the more beneficial the impact of nature [10].

Being exposed to nature in neighborhoods is hypothesized to decrease loneliness by encouraging social interaction and helping to create crucial social relationships that buffer stress [11]. For parents, neighborhood parks are associated with increased children’s play and social support between mothers and non-family members [12]. Exposure to neighborhood parks has been associated with an increase in moderate physical activity [13] which, in turn, reinforces ongoing park visits and is associated with decreased stress [14]. Exposure to nature in parks also increases emotional attachment to the broader concept of nature. We refer to this emotional attachment as nature affinity—which has been associated with pro-environmental behavior and ongoing park visits [15].

Parks provide the most readily available access to nature for many individuals living in urban areas [12, 16]. As a result, clinicians have been called upon to create community partnerships with parks [17, 18]. Termed park prescriptions, these partnerships are growing in number [19], yet there remains little rigorous clinical research to guide the practice. There are many studies showing an association between nature exposure and improved stress; fewer studies are experimental, and very few are randomized, controlled, or clinical trials. Among the few controlled studies are retrospective studies (such as a 1991 study by Ulrich and colleagues of improved surgical recovery with nature exposure) [20], those conducted within a laboratory, and several that measured cognitive outcomes immediately before and after a walk through a park versus less natural settings (neighborhoods, urban streets) [21–23]. Most study participants in clinical research regarding parks and health to date have been healthy and male, though hypertensive patients [24] and the elderly have also been investigated [25]. A well-described clinical intervention with clear understanding of screening guidelines, duration and intensity of the intervention, and clinically relevant outcome measures has not been undertaken. Implementation science is especially lacking in low-income clinics where those served also often have the least access to nature and may encounter multiple levels of barriers to outdoor leisure time [26, 27].

As the site of a standing park prescription program, our pediatric primary care clinic had the unique opportunity to address these research gaps. Since 2012, we have partnered with our local park agency to reduce barriers to accessing nature for the health of pediatric patients and their families. Our park prescription program consisted of recommendations by a physician to be in nature as well as enrollment in monthly family outings into nature with food, transportation, and programing provided. In its third year, with more than 1,000 patient visits to parks, we were poised to evaluate the efficacy of this park prescription program as a clinical intervention.

The Stay Healthy In Nature Everyday (SHINE) study was conducted in order to test if park prescriptions will improve stress and other behavioral and health outcomes for parents at a low-income clinic and if a supported park prescription, in the form of physician counseling, a map of local parks, journal, and a pedometer with group park outings for families will have a greater impact on stress and other outcomes in low-income parents than the same park prescription with no group visits.

## Methods

### Participants, recruitment and randomization

SHINE was a randomized trial conducted at a Primary Care Clinic (PCC) that is a Federally Qualified Health Center (FQHC) and serves a linguistically, racially and culturally diverse group of pediatric patients living near the federal poverty level in Oakland, California. Recruitment was done by referrals from health care providers and in the clinic waiting room. Parent-child pairs were eligible if the child was a clinic patient between 4–18 years old and the guardian was at least 18 years of age. Parent-child pairs were excluded if either was concurrently enrolled in a weight loss or exercise program, unable to walk or be physically active, or unavailable for the park outings and two follow-up visits over three months. The study was designed to measure stress in adult parents of children accessing a pediatric safety-net hospital, and the sample size was calculated to detect a difference in parental stress measured by the PSS10 instrument [28]. A detailed description of the sample size calculation and rationale for enrollment of both parent and child have been published in a previous publication of the SHINE study methods [28]. Here we present findings from adult participants; a secondary data analysis of pediatric and dyad data will be presented separately.

After enrollment, baseline data were gathered. All enrollees then received counseling regarding the potential health benefits of time in nature. Parents received a postcard, which included a map and bus routes to seven local parks, a journal to document their visits, and a pedometer The postcard listed the following benefits of time in nature, including:

1. Walking in nature keeps you active and strong.
2. In nature, you experience less stress and anxiety and increase your sense of well-being.
3. Time outside in nature with family and friends reduces loneliness.
4. Nature helps create a sense of wonder and can make your mind sharper.

Parents were also instructed to use the pedometer to record steps at baseline and park visits in the week prior to each follow up visit. The principal investigator, a pediatrician, reviewed the benefits of time in nature and gave each participant a park prescription using the following script:

“I recommend that you are outside, in nature, three times a week, for at least one

hour at a time; here is a map with several local parks where you can find nature.”

After the counseling was completed, child-parent pairs were randomized to one of two parallel treatment groups using REDCap hosted at University of California, San Francisco (Research Electronic Data Capture Consortium, Bethesda, Maryland, USA) [29]. Randomization was performed with a one to two ratio given the clinic’s one-year pilot experience, which showed that less than half of those families who received a park prescription attended the group outings. Random permuted blocks were used, and we stratified by child’s gender. There was no blinding of participants as to which intervention they received.

#### Independent park prescription group

The independent park prescription group received counseling by a pediatrician about nature according to the script above, the postcard with a map of local parks, journal, pedometer, and no further intervention after randomization.

#### Supported park prescription group

Parents randomized to the supported park prescription group received counseling by a pediatrician about nature according to the script above, a postcard with the map of local parks, journal, and pedometer. After randomization, they were advised to attend group nature outings on three consecutive Saturdays, and were invited to bring their families. Participants received phone reminders on the Wednesday before outings and a text on the Friday before the Saturday outing. On the day of the outing participants arrived at the clinic where they met the physician, the research assistant, several hospital volunteers, and other enrolled families. A bus transported the group to three local parks consecutively over the three weeks: a bayfront park with a beach, a lake with woodlands, and a redwood forest. Each park is located within 15 minutes of the clinic by vehicle or public transportation. At the park, participants were greeted by park staff, played games, picnicked, had unstructured play, and a light walk. Outings concluded with quiet reflection and an opportunity to share experiences before a bus took participants back to the clinic. The study was conducted in three recruitment periods waves in order to keep the group number to 30–50 people per outing (this number includes enrollees and non-enrollee family members).

The study was approved by UCSF Benioff Children’s Hospital Oakland Institutional Review Board on July 17, 2015; all participants provided informed consent and were free to terminate participation at any point. Patient recruitment occurred between July 21, 2015, and September 23, 2017. Patient follow-up after one month and three months. The authors confirm that all ongoing and related trials for this drug/intervention are registered. This trial was registered with clinicaltrials.gov on 7/17/15, and was released on 11/7/2015 due to a delay.

### Variables

**Fig 1:**
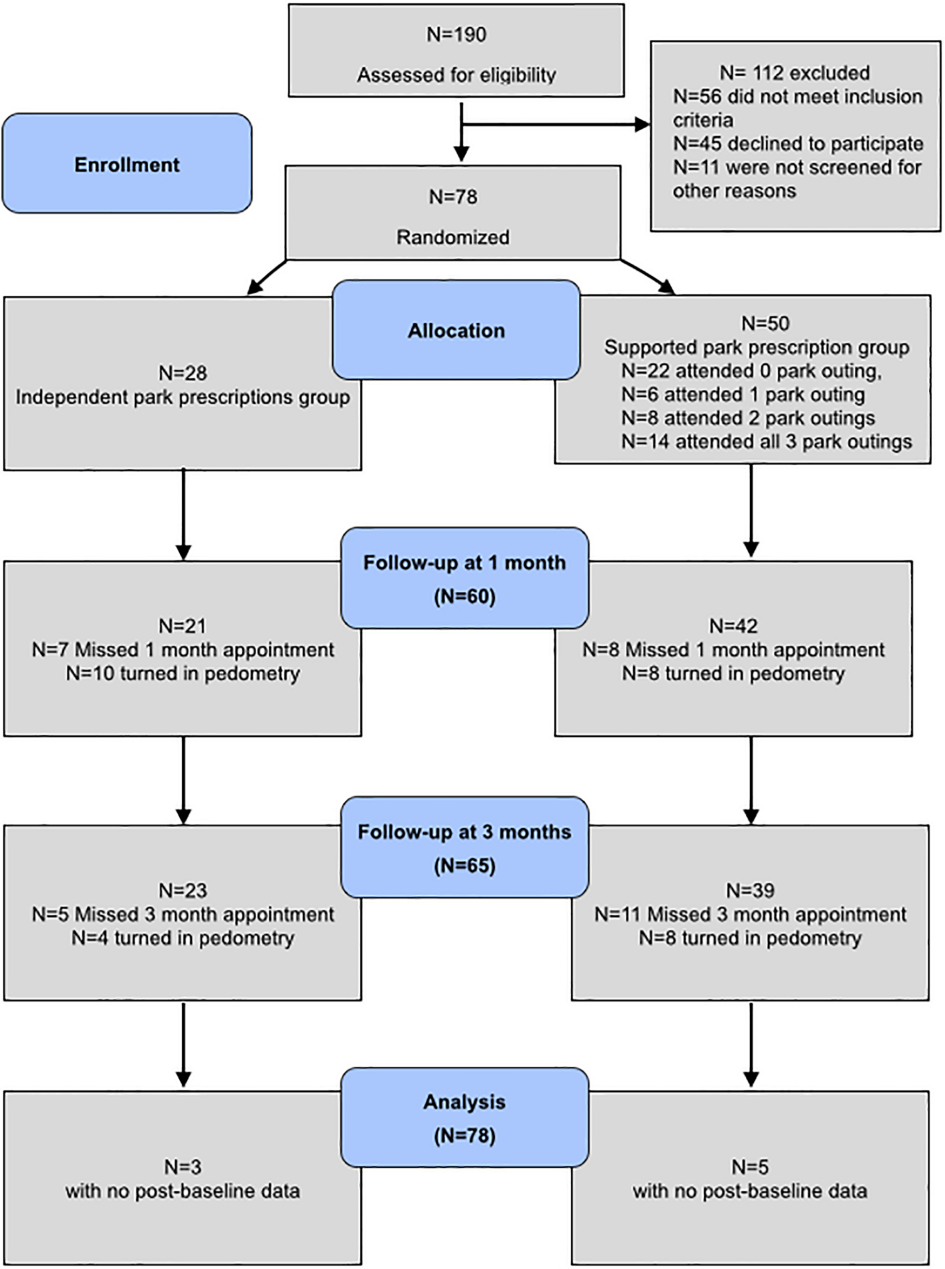
CONSORT flow diagram showing the progress of patients throughout the Stay Healthy In Nature Everyday (SHINE) trial.

**Fig 2:**
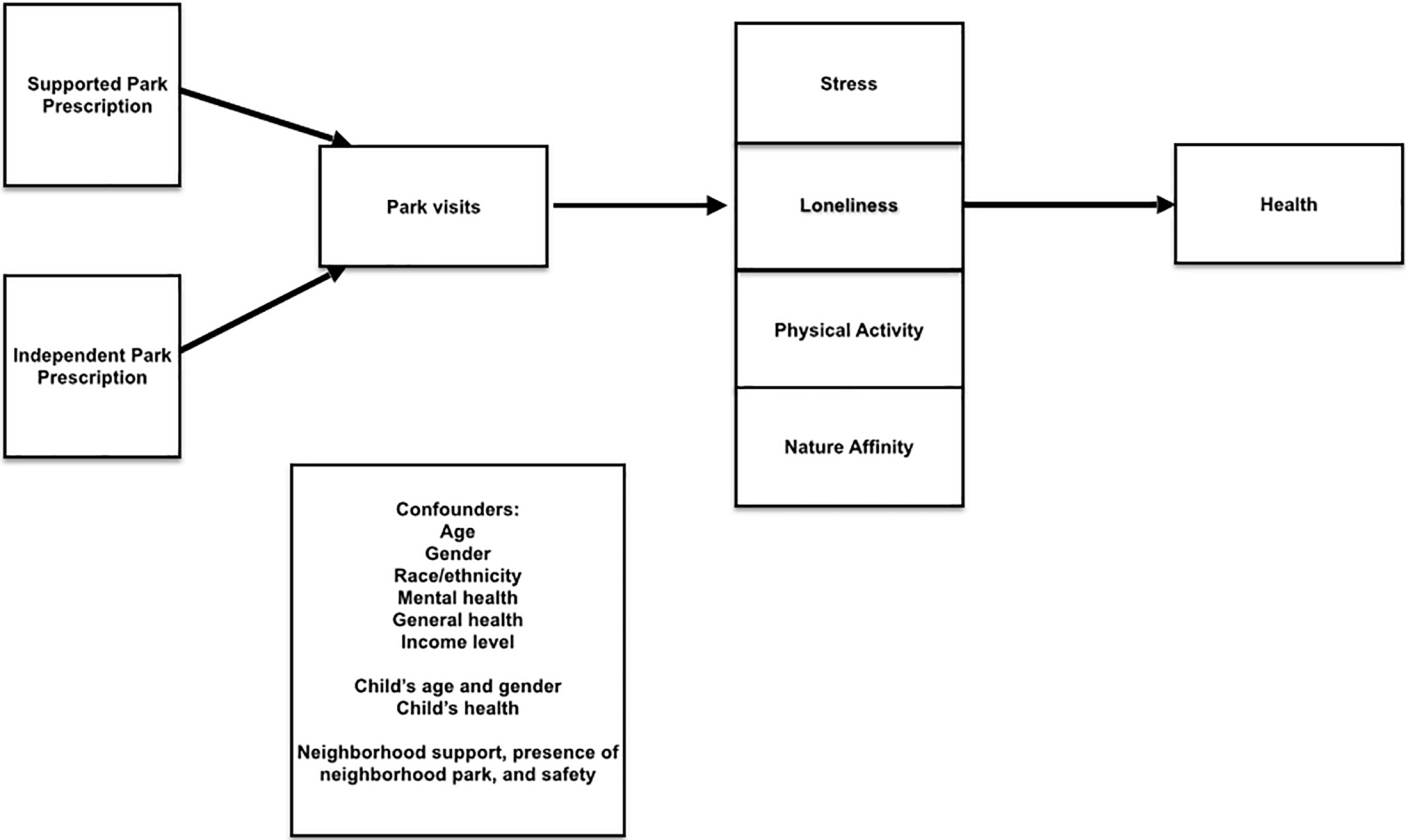
Conceptual model guiding variable measurement during the Stay Healthy In Nature Everyday (SHINE) Trial.

Measures occurred in both groups at zero, one, and three months after enrollment (Fig 1). Incentives for the enrollment and follow-up visits were $20, $20, and $40 and the same in both groups. Outcomes and confounders were chosen a-priori based on previously published research [30] and are summarized in Fig 2.

Our primary outcome stress was measured using the Perceived Stress Score (PSS10) [31, 32], a 10-item validated Likert scale, with responses to each item with five options (i.e., never, almost never, sometimes, usually, always).

We also assessed five secondary outcomes. Park visits per week were measured by parent recall of the number of times they visited a park in the seven days prior to each of their follow-up visits. Participants were able to consult their journals for number of park visits and physical activity, a method with established reliability and validity [33].

We measured loneliness using the validated modified UCLA Loneliness Score [34], a three-item Likert scale: “How often do you feel that you lack companionship?”, “How often do you feel left out?” and “How often do you feel isolated?” with three response options: hardly ever, some of the time, often.

We assessed physical activity via self-report. Participants were asked: “How many minutes of moderate activity have you engaged in on an average day over the last week? Moderate activities refer to activities which make you break a sweat, but wouldn't prevent you from carrying on a conversation. Examples include walking briskly or actively playing with children.” The physical activity self-report measure was created by the authors based on guidelines from the Centers for Disease Control and Prevention and have not been validated [35].

Enrollees were asked to monitor their pedometry in the week prior to their first outing and in the week prior to each follow up appointment by wearing Accusplit Eagle 170 DigiWalker 2 pedometers (Accusplit, Inc., Livermore, California, USA) at all times except when sleeping, bathing, or swimming, as well as to complete a brief activity log for two weekdays and one weekend day per week, a validated procedure [36].

We measured physiologic stress using salivary cortisol. During the pilot phase, we determined that compliance was low when patients were asked to bring salivary samples in from home, and, as a result, study staff obtained the specimens during clinic visits with an attempt made at obtaining the saliva samples at the same time of day at baseline and follow-up. Salivate (Sarstedt, Rommelsdorf, Germany) collection devices were used to obtain saliva samples.

We measured nature affinity using a 15-item validated scale which has strong correlation to outdoor behavior and strong construct validity established [15].

Covariates recorded included socio-demographics, such as parent’s age, gender, family income level (percent of federal poverty level), race, ethnicity, child’s age and child’s gender. Clinical variables were parents’ mental and physical health, and the presence of chronic illness in the enrolled child such (ADHD, depression, anxiety, autism spectrum disorder, developmental delay, asthma, obesity, or other).

We assessed neighborhood social support by summing the responses to four validated items including “People in my neighborhood help each other out”; “We watch out for each other's children in this neighborhood”; “There are people I can count on in this neighborhood”; and “If my child were outside playing and got scared or hurt, there are adults nearby who I trust to help my child.” These questions, as an aggregated sum, have been previously correlated with mental health and stress [37]. We asked participants to rate how safe they felt their child was in their community or neighborhood and whether or not there was a park in their neighborhood, as park access has also been correlated with stress [38].

### Statistical analysis

To assess whether park prescriptions improved stress and other outcomes for parents, we compared mean values for each outcome for the combined groups at baseline and three months using a paired t-test.

To examine the effect of group assignment on stress and secondary outcomes for parents, we compared the difference in mean scores within treatment groups using independent t-tests.

We performed multivariable analysis to examine the estimated effect of group assignment on the primary outcome (PSS10 score), while accounting for those baseline imbalances as well as potential correlates that could be associated with the outcome over time. We fit models with a generalized estimating equation using an autoregressive correlation structure and robust variance estimators [39]. The final multivariable model included group assignment and all covariates that maintained an association with PSS10 at a threshold of p<0.05.

Prior to analyses, outliers for the cortisol variables were winsorized at three standard deviations. In addition, all cortisol variables underwent a natural log transformation to correct for deviations from normality (i.e., skewness < 2 and kurtosis < 7) [40]. The natural log transformed cortisol variables were within an acceptable range for skewness and kurtosis and were used.

The analysis presented is based on intention to treat; all participants randomized were included in the analysis unless otherwise noted. All analyses were conducted using Stata SE, version 10.1 for Macintosh (Stata Corporation, College Station, TX).

## Results

We randomized 78 child-parent pairs, 50 in the supported group and 28 in the independent group (Fig 1). Of the enrolled parents, 68 (87%) were women, the mean age was 38, 95% were non-white, and the majority (68%) lived below 200% of the federal poverty line. Of the enrolled children, 40 (51%) were male, with average age of 8.8 years (SD 3.1 years), and 59 (76%) of the children had at least one chronic illness. Participants in the supported and independent park prescriptions groups did not differ significantly except for self-reported mental health by parents, which was rated poorer in the supported park prescriptions group (p = 0.01) (Table 1). Overall study completion rate was high (10% lost to any follow up; Fig 1). Of 50 enrollees in the supported park prescription group, 56% attended at least one outing and 44% attended two or more. Because few participants turned in their pedometry recordings (23%; Fig 1) these data were not analyzed.

**Table 1:** SHINE parents: Baseline demographics, family, community, and outcome measures.

		N		Independent		Supported		p-value
		N	%	N	%	N	%	
Total		78	100	28	36%	50	64%	
Gender								0.16*
	Female	68	87%	22	79%	46	92%	
Age								0.95***
	18–34	29	37%	11	39%	18	36%	
	35–44	28	36%	10	36%	18	36%	
	45 and older	21	27%	7	25%	14	28%	
Race/Ethnicity								0.37*
	African American	52	67%	20	71%	32	64%	
	Non-Latino White	4	5%	1	4%	3	6%	
	Latino	12	15%	2	7%	10	20%	
	(1) Other	10	13%	5	18%	5	10%	
Percent Federal Poverty (4 missing)								0.55*
	<100% FPL	11	14%	3	11%	8	16%	
	100–199% FPL	42	54%	14	50%	28	56%	
	200% or more FPL	12	15%	7	25%	5	10%	
	400% or more FPL	9	12%	3	11%	6	12%	
Education Level (2 missing)								.35*
	No high school degree	12	15%	2	7%	10	20%	
	High school graduate	50	64%	21	75%	29	58%	
	College graduate	14	18%	4	14%	10	20%	
Primary Language								.16*
	English	62	79%	21	75%	41	82%	
	Spanish	7	9%	1	4%	6	12%	
	Arabic	3	4%	2	7%	1	2%	
	(2) Other	6	8%	4	14%	2	4%	
Country of birth (1 missing)								1*
	United States	64	82%	23	82%	41	82%	
	Not United States	13	17%	5	18%	8	16%	
Health (Self-report)								0.195*
	Poor or fair	40	51%	10	35%	30	60%	
	Good	27	35%	12	43%	15	30%	
	Excellent or Very good	11	14%	6	21%	5	10%	
Mental Health (Self-report)								0.01*
	Poor or fair	34	44%	11	39%	23	46%	
	Good	25	32%	5	18%	20	40%	
	Excellent or Very good	19	24%	12	42%	7	14%	
Neighborhood support score mean (0–16 scale) Mean and (SD)		11.9 (4.8)		11.8 (5.2)		12 (4.6)		0.83**
Neighborhood safe for child (1 missing)								1.0*
	Never or sometimes	38	49%	14	50%	24	48%	
	Usually or Always	39	50%	14	50%	25	50%	
Access to a park								0.7*
	Strongly disagree or disagree	19	24%	6	21%	13	26%	
	Neither agree nor disagree	4	5%	2	7%	2	4%	
	Agree or strongly agree	55	71%	20	71%	35	70%	
Baseline Measures		N Mean(SD)		Independent N Mean (SD)		Supported N Mean (SD)		p-value
PSS-10 scores (0–40 unit scale)		78	19.0 (7.0)	28	17.3 (7.8)	50	19.9(6.6)	0.12**
Park visits in previous week (park visits reported/week)		78	1.7 (2.0)	28	1.7 (2.2)	50	1.7(1.9)	0.97**
Loneliness scores (0–9 unit scale)		78	5.6 (1.7)	28	5.3 (1.9)	50	5.7 (1.7)	0.33**
Minutes of moderate physical activity in past week (minutes per week)		78	36.3 (28.9)	28 39.4(28.9)		50 34.6(29.0)		0.48**
Physiologic stress (Cortisol level in ug/dL)		71	0.2(0.1)	26	0.2(0.07)	45	0.2(0.1)	0.55*
Nature Affinity (1 to 7 unit scale)		78	5.4 (1.20)	28	5.53(1.08)	50	5.33(1.27)	0.49**

(1) Native American, Middle Eastern, API

(2) Nepali, Tongan, Mandinca, Fulanis, Ahmaric, French, Farsi

* Fisher exact

** Independent t-test, 2 tailed

*** Chi-squared test

The combined study groups showed a significant decrease in stress and improvements in secondary outcomes over time for all participants when analyzed as a group (Table 2). Over the three-month course of the study, stress for all participants dropped 1.71 points on the 40-point PSS10 scale (95% CI -3.15, -0.26). Park visits increased by 1.22 visits a week (95% CI 0.57, 1.86). Loneliness decreased by 1.03 points on a nine point scale (95% CI -1.52, -0.54); participants reported an approximately 24 minute increase in moderate physical activity per week (95% CI 11.05, 36.82); cortisol level decreased by 0.05 ug/dL (95% CI -0.09, -0.02), and nature affinity increased by 0.35 points on a 6 unit scale (95% CI 0.05, 0.65).

**Table 2:** Changes in primary and secondary outcomes for all participants from baseline to three months.

			Baseline		3 month					
		N	Mean	SD	Mean	SD	Difference		95% CI of Mean Diff	p
**Primary Outcome**										
	**Stress PSS-10 score (0–40 unit scale)**	65	18.83	7.36	17.12	5.83	-1.71		[-3.15, -0.26]	0.0212
**Secondary Outcomes**										
	**Park visits in previous week (park visits reported/week)**	65	1.58	1.87	2.80	2.29	1.22		[0.57, 1.86]	0.0004
	**Loneliness (0–9 unit scale)**	64	5.42	1.75	4.39	1.69	-1.03		[-1.52, -0.54]	0.0001
	**Minutes of moderate physical activity in past week (minutes per week)**	63	34.57	29.35	58.51	43.58	23.94		[11.05, 36.82]	0.0004
	**Physiologic stress (Cortisol level in ug/dL)**	55	0.18	0.13	0.12	0.07	-0.05		[-0.09,-0.02]	0.0008
	**Nature Affinity (1 to 7 unit scale)**	65	5.40	1.20	5.76	1.10	0.35		[0.05, 0.65]	0.0241

The change in PSS10 did not differ significantly between the supported and independent park prescription groups at either one-month or three-month follow up. The independent park prescription group had a non-significant and 0.91 point larger drop in mean PSS10 score between baseline and one month (95% CI -4.47, 2.65) (Table 3). At three months, there was no significant difference between groups; the supported park prescription group had a non-significant and 0.36 larger (95% CI -2.69, 3.40)) drop in mean PSS10 score when compared to the independent park prescription group (Table 4).

**Table 3:** Changes between baseline and 1-month follow-up in study outcomes by to group assignment, SHINE trial.

Measure	Group Assignment	N^(1)^	Baseline Mean (SD)	1 Month Mean (SD)	^(2)^Within group changes [95% CI]	Δ supported–Δ independent [95%CI]	^(3)^p
**Stress PSS-10 score (0–40 unit scale)**	Independent	21	18.00 (8.26)	15.19 (6.52)	-2.81 [-6.51, 0.89]	-0.91 [-4.47, 2.65]	0.6099
	Supported	39	20.00 (6.98)	18.10 (5.59)	-1.90 [-3.70, -0.09]*		
**Park visits in previous week (park visits reported/week)**	Independent	21	1.67 (2.15)	3.48 (2.58)	1.81 [0.82, 2.80]*	0.73 [-0.60, 2.06]	0.2751
	Supported	39	1.62 (1.74)	2.69 (2.27)	1.08 [0.24,1.92]*		
**Loneliness (0–9 unit scale)**	Independent	20	5.65 (1.87)	4.40 (1.54)	-1.25 [-2.09, -0.41]*	-0.22 [-1.20, 0.76]	0.6502
	Supported	37	5.57 (1.67)	4.54 (1.35)	-1.03 [-1.61, -0.45]*		
**Minutes of moderate physical activity in past week (minutes per week)**	Independent	18	43.28 (23.87)	64.00 (39.05)	20.72 [-4.96, 46.41]	-10.05 [-46.53, 26.45]	0.5834
	Supported	38	34.37 (28.98)	65.13 (59.96)	30.76 [8.28, 53.25]*		
**Physiologic stress (Cortisol level in ug/dL)**	Independent	17	0.13 (0.05)	0.12 (0.06)	-0.01 [-0.04,0.03]	-0.02 [-0.13, 0.09]	0.7105
	Supported	35	0.17 (0.14)	0.19 (0.18)	0.01 [-0.07, 0.09]		
**Nature Affinity (1 to 7 unit scale)**	Independent	21	5.72(0.94)	5.93 (1.17)	0.21 [- 0.18, 0.611]	-0.27 [-0.83, 0.30]	0.3458
	Supported	39	5.31(1.33)	5.79(1.13)	0.48 [0.11, 0.85]*		

1) Patients with baseline and one month measures completed are included in this analysis.

2) Within group difference from baseline to one month.

3) P-values derived from independent t-test of the difference in mean scores within the independent group compared to the difference in means scores in the supported group.

* Associated p-value < 0.05.

**Table 4:** Changes between baseline and 3-month follow-up in study outcomes in by group assignment, SHINE trial.

Measure	Group Assignment	N^(1)^	Baseline Mean (SD)	3 Month Mean (SD)	^(2)^Within group changes [95% CI]	Δ supported–Δ independent [95%CI]	^(3)^p
**Stress PSS-10 score (0–40 unit scale)**	Independent	23	17.30 (8.21)	15.83 (6.91)	-1.48 [-4.55, 1.60]	0.36 [-2.69, 3.40]	0.8164
	Supported	42	19.67 (6.81)	17.83 (5.11)	-1.83 [-3.42, -0.25]*		
**Park visits in previous week (park visits reported/week)**	Independent	23	1.56 (2.09)	3.91 (2.45)	2.35 [1.32, 3.37]*	1.75 [0.46, 3.04]	0.0085
	Supported	42	1.60(1.77)	2.19 (1.98)	0.60 [-0.20, 1.39]		
**Loneliness (0–9 unit scale)**	Independent	22	5.09 (1.87)	4.32 (1.70)	-0.77 [-1.48, -0.06]*	0.39 [-0.65, 1.44]	0.4531
	Supported	42	5.59 (1.68)	4.43 (1.70)	-1.17 [-1.84, -0.50]*		
**Minutes of moderate physical activity in past week (minutes per week)**	Independent	22	39.77 (28.85)	73.68 (48.19)	33.91 [8.20, 59.62]*	15.32 [-11.05, 36.82]	0.2604
	Supported	41	31.78 (29.59)	50.37 (39.11)	18.58 [3.75, 33.42]*		
**Physiologic stress (Cortisol level in ug/dL)**	Independent	21	0.16 (0.11)	0.12 (0.07)	-0.05 [-0.10, 0.00]	0.01 [-0.06, 0.08]	0.9057
	Supported	34	0.18 (0.14)	0.13 (0.07)	-0.06 [-0.11, -0.01]*		
**Nature Affinity (1 to 7 unit scale)**	Independent	23	5.54 (0.98)	5.62 (1.43)	0.08 [-0.40, 0.55]	-0.42 [-1.04, 0.19]	0.1749
	Supported	42	5.33 (1.31)	5.84 (0.86)	0.50 [0.11, 0.90]*		

1) Patients with baseline and three months measures completed are included in this analysis.

2) Within group difference from baseline to three months.

3) P-values derived from independent t-test of the difference in mean scores within the independent group compared to the difference in means scores in the supported group.

* Associated p-value < 0.05.

**Fig 3:**
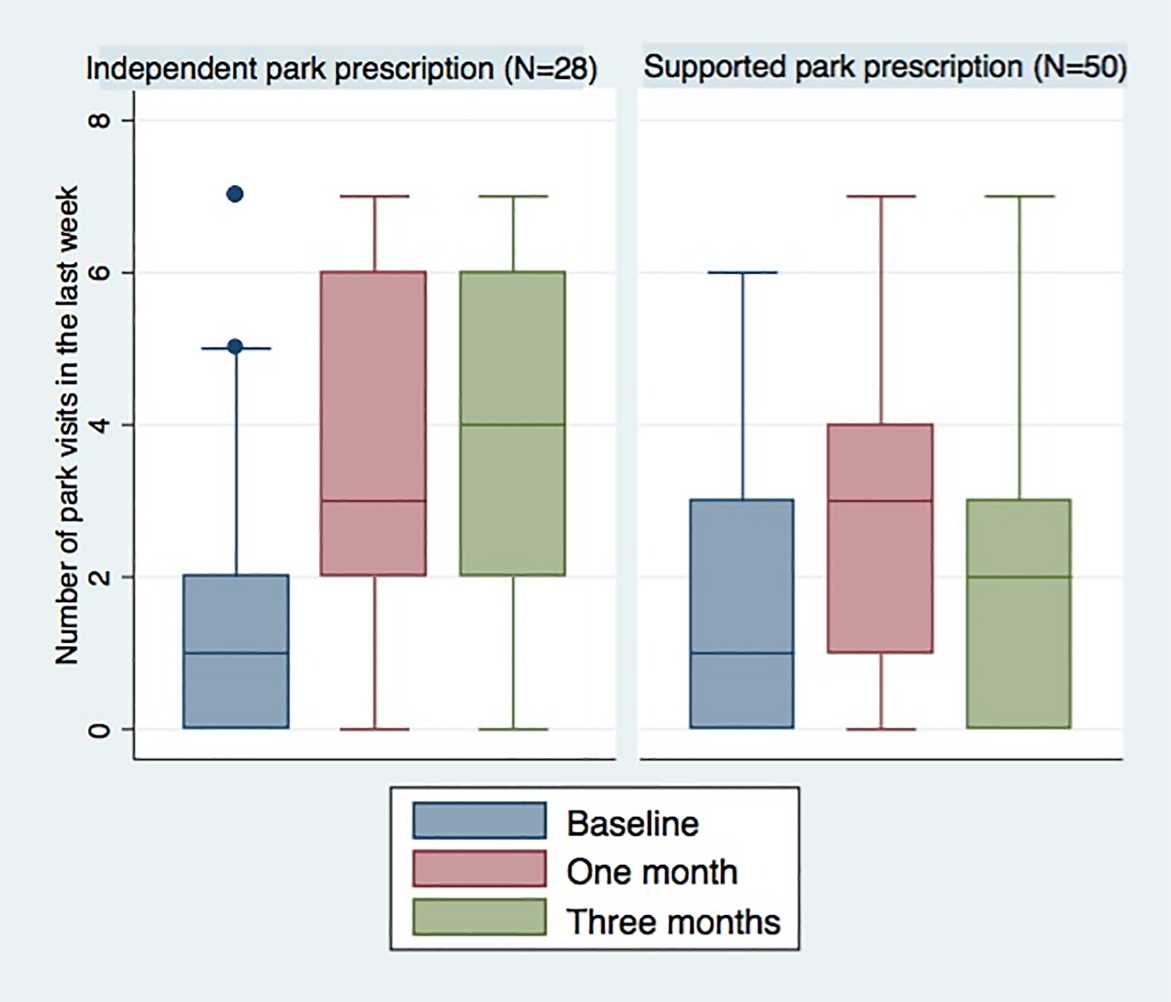
Number of park visits per week by treatment group. Legend: Median, interquartile range, and outer values are shown for each measurement point.

All secondary outcomes showed no significant group difference over time (Tables 3 and 4), except for park visits between baseline and three months. The independent park prescription group had a statistically significant increase of 1.75 park visits per week compared to the supported park prescription group [0.46, 3.04] (Table 4 and Fig 3). The supported park prescription group had a greater increased in mean nature affinity, although this difference of 0.42 on the 6 unit scale was not a significant difference [-1.04, 0.19].

The final longitudinal multivariable linear model showed group visits were associated with a decrease in PSS10 score, although this finding was not statistically significant (-0.13 group mean difference, 95% CI -2.67, 2.42). Poorer parental mental health was associated with higher stress. Male gender in the parent was associated with improved PSS10 score over the course of the study, and this association was significant (Table 4). Each unit increase in park visits per week during the study was significantly associated with a 0.5 point decrease in stress over the three-month study (-0.53 group mean difference, 95% CI -0.89, -0.16; p = 0.005) (Table 5).

**Table 5:** Estimated effects of group assignment on PSS10 over baseline, one, and three months with potential confounders of parental mental health, gender, and park visits on parent stress.

Outcome N Observations	Parameter	Effect Size	95% CI	p
**Stress** **N = 60** **175 observations**				
	Supported group	- 0.13	[-2.67, 2.42]	0.923
	Male gender in parent	- 4.95	[-8.27, -1.62]	0.004
	Parent mental health ^(1)^	1.44	[0.41, 2.48]	0.006
	Park visits per week	- 0.53	[-0.89, -0.16]	0.005

(1) Parent mental health defined as an ordered categorical variable. Participants ranked their mental health from excellent to poor and the responses were transformed to a 1 to 5 scale, with a higher number signifying worse mental health.

## Discussion

In this prospective clinical trial of 78 low-income parents, we report a significant decrease in stress, as well as improvement in park visits, loneliness, physical activity, physiologic stress and nature affinity over the three months of the trial. Our study showed that park prescriptions are a promising tool for addressing stress in low-income parents, a population at high need for community-based supports for stress. Future research should include a controlled trial comparing a park prescription as we have described to no intervention.

In this randomized trial, supports beyond a physician’s recommendation, a map of local parks, journal, and pedometer, in the form of facilitated group parks outings for families, did not have additional impact on decreasing stress relief and loneliness or increasing physical activity, physiologic stress, and nature affinity. This study raises important questions as to the role of clinics in leading park outings. There are many studies measuring the effects of nature immediately before and after a single and facilitated nature outing such as a walk through a forest for 20–90 minutes [22, 23]. SHINE is one of the first to follow patients for several months after a nature outing. Our findings suggest that the model of a single, occasional nature outing, even if several hours long, is insufficient for long term impact. Others have suggested that longer (on the order of three days) and more immersive nature experiences (in wilderness) are necessary to show the maximal effect on brain function and creativity [41]. If so, cost-effectiveness studies would be necessary to examine the value of interventions that require larger wilderness excursions.

Our study suggests that for some patients, behavioral counseling by a primary care provider with tools to find and experience parks were adequate for encouraging park going behavior. Independent park prescriptions resulted in higher increases in park visits than supported park prescriptions, and each increase in weekly park visits was associated with an incremental decrease in stress. Perhaps it is exactly because parks are freely accessible and convenient for families facing many economic and social constraints that a park prescription is more effective when left open ended. Future research can delineate who benefits from an open-ended prescription and who may benefit from clinical supports. Future studies should explore the circumstances facing parents, such as language spoken or family structure, in deciding whether or not to visit parks. Finally, it will be useful to describe park variables such as the type of nature available and whether families are comfortable there, the different park staff available in gender, age and presence of a uniform and whether that had any impact on participation and outcome.

Over the course of our study, and regardless of group assignment, each increase in weekly park visits reported was associated with incremental and small improvement in stress. This finding, that parents had improved stress with increased weekly park visits whether or not those visits were facilitated group outings, reinforces the potential efficacy of park prescriptions in reducing stress, and suggests that independent park going may be the causal mediator. The fact that we found that the health benefit associated with each park visits was small and incremental supports others who have argued that frequent, daily doses of nature may be most useful in improving stress and other health outcomes [42]. Our study reinforces those who have suggested that nature, not only that in a park, but also trees & vegetation around residences, greenways, green vacant lots, gardens, school yards, neighborhood trees, and local woodlands, are as important to health as the presence of a park [43].

Weekly park visits remained significantly associated with stress while physical activity did not on multivariable analysis. This may be may be because we did not designate whether the reported physical activity was indoors, outdoors, or in nature. Others have demonstrated some added mental and emotional health benefits to exercising out of doors in nature as compared to indoors. In a review of 25 studies comparing the physical activity conducted in a human-made setting (such as inside a gymnasium), to the same activity conducted in a natural setting, activities in a natural environment resulted in reduced negative emotions (e.g., anger, fatigue, sadness) as well as improvements in attention [44]. Blood pressure and cortisol showed less consistent changes, but the studies conducted to date have small sample sizes and heterogeneity in target population and in how they have defined their outcome [44]. It is also of note that while the association between nature and stress has been clear, the association between nature and physical activity has not. Being outdoors in natural settings can also involve meaningful but sedentary recreation activities such as picnicking with friends or family, or taking a moment to relax [45]. These activities have mental health and stress-reduction benefits that may have affected our outcome. Future studies can further investigate the different contribution of physical activity versus nature exposure on stress, and effective ways to measure both in a busy clinic setting.

On longitudinal analysis, poorer mental health was an independent predictor of increased stress. Park prescription programs should take into account the barriers faced by high health needs populations, including those with poor mental health. Baseline mental health has been correlated with response and retention in programs aimed at parents [46]. Male gender was correlated with improved stress. Other studies have also shown a disparity between women and men in the stress response to nature-experiences and have suggested that women need a longer exposure to nature to gain a measurable stress reduction [47].

This study has limitations. The lack of a control group that received no park prescription at all means that the groups may have been too similar to detect a between group difference in outcomes over time. Future research should have a three-arm design (park prescription, supported park prescription, and no-intervention control) to fully address this question. The parks available to this clinic included a bayfront, a lake, and a forest, areas not accessible to all urban primary care clinics. The study results, therefore, may not be generalizable to other contexts, climates, or populations, or to moderate and high-income families. The SHINE intervention has an inherent social component to the visits to the park; it is not possible to separate the social from nature factors in the intervention. Finally, our study was underpowered to detect small group differences.

Despite these limitations, SHINE is one of the first prospective clinical studies, and the first randomized trial, that has tested whether a clinical intervention encouraging visits to nature can improve stress, increase park visits, decrease loneliness, improve moderate physical activity, physiologic stress, and nature affinity. Like other low-income populations, the patients in this sample reported an average level of stress that was about one standard deviation higher than the national average [48]. This study is the first to look at nature affinity as a health outcome. Further research can help understand whether a clinicians office can help encourage human relationships with natural spaces and the development of environmental altruism.

Our study suggest that the clinician-patient relationship is an important one in encouraging outdoor time for stress relief. Like others, we have identified primary care visits as an important opportunity to screen for social determinants of health such as stress [49]. Our study findings suggest that a park prescription may be of benefit in addressing stress and that extensive behavioral supports, beyond a clinician’s recommendation and maps of local parks, a journal, and pedometer may not be necessary to encourage park visits in low income families. A randomized controlled trial of park prescriptions, which includes attention to issues of adherence, dose, duration of exposure, and length of effect, that examines how to maximize the effectiveness of provider-based counseling for time in nature and the cost-efficacy of parks prescriptions is an opportunity for ongoing research.

## Supporting information

S1 File CONSORT checklist. (DOCX)

S2 File Data set. (XLS)

S3 File IRB protocol. (DOCX)
