# Supplementary material for: Effect of park prescriptions with and without group visits to parks on stress reduction in low-income parents: SHINE randomized trial
Source: PLoS One. 2018 Feb 15;13(2):e0192921. doi: 10.1371/journal.pone.0192921 (PMC5814008; doi:10.1371/journal.pone.0192921)

Institutional Review Board

Application for Study Review

(The most current version of this application is available online at [www.chori.org/Clinical_Studies/IRB/IRB_Forms.html](http://www.chori.org/clinical_studies/irb/irb_forms.html))

| Study Information | | |  |
| --- | --- | --- | --- |
|  | **IRB Number:** | 2015-042 | |
|  | **Protocol Title:** | SHINE (Stay Healthy In Nature Everyday) | |
|  | **Principal Investigator:** | Nooshin Razani, MD MPH | |
|  | Department: | Children’s Hospital and Research Center | |
|  | Address (if outside CHRCO): | 5220 Claremont Ave | |
|  | Phone: | (415 )  722  - 1915 | |
|  | Fax: | (510) 547 - 2702 | |
|  | E-mail: | [nrazani@mail.cho.org](mailto:nrazani@mail.cho.org) | |
|  | **Co-Investigator:** | Kelley Meade, MD | |
|  | Department: | Ambulatory Care | |
|  | Address (if outside CHRCO): | 5220 Claremont Ave | |
|  | Phone: | ( 510 )  428 - 3885 ext. 2793 | |
|  | Fax: | (510) 547 - 2702 | |
|  | E-mail: | [kmeade@mail.cho.org](mailto:kmeade@mail.cho.org) | |
|  | **Co-Investigator:** | Mindy Benson, MSN PNP | |
|  | Department: | Ambulatory Care | |
|  | Address (if outside CHRCO): | 5220 Claremont Ave | |
|  | Phone: | 510-428-3885 ext. 4145 | |
|  | Fax: | (510)  547 - 2702 | |
|  | E-mail: | [mbenson@mail.cho.org](mailto:mbenson@mail.cho.org) | |
|  | **Study Coordinator:** | Christine Schudel | |
|  | Phone: | (  510   ) 428 - 3885 ext. 3226 | |
|  | Fax: | (510 )  547 - 2702 | |
|  | E-mail: | [cschudel@mail.cho.org](mailto:cschudel@mail.cho.org) | |
|  | **Primary Contact Person:** | Nooshin Razani, MD MPH | |

|  | Funding | | | |
| --- | --- | --- | --- | --- |
|  | Federal Industry (e.g. Pharmaceutical or Device Company Private Foundation  Internal Grant Program Children's Oncology Group (COG) Other, Specify: | | | |
|  | **Funding Source:** | | East Bay Regional Parks District and Foundation | |
|  | **Contract or Grant Title:** | |  | |
|  | **Contract or Grant #:** | |  | |
| **Address of Sponsor:** | | 2950 Peralta Oaks Ct  Oakland, Ca | |  |
|  | |  |  |  |
|  | **Contact Person:** | | Carol Johnson, Assistant General Manager  Mona Koh, Community Relations Manager | |
| **Phone:** | | ( 510 )  544 - 2003  (510) 544-2216 | |  |
| **E-mail:** | | [cjohnson@ebparks.org](mailto:cjohnson@ebparks.org), [mkoh@ebparks.org](mailto:mkoh@ebparks.org) | |  |

| Subject Category | |
| --- | --- |
|  | Subjects admitted strictly for research purposes. Hospitalization and laboratory costs are paid by the funding source. |
| X | Research subjects receiving established medical care. Hospitalization and laboratory costs paid by third party. (except for tests performed exclusively for the study) |
|  | Research subjects admitted on an industry-sponsored protocol. All costs paid by industry sponsor. Requires $2,200 IRB application and set-up fee. |

| ClinicalTrials.Gov Registration | |
| --- | --- |
| Does this study need to be registered on [www.clinicaltrials.gov](http://www.clinicaltrials.gov)? (see below) | No x Yes |
| General Requirements [U.S. Public Law 110-85](http://frwebgate.access.gpo.gov/cgi-bin/getdoc.cgi?dbname=110_cong_public_laws&docid=f:publ085.110.pdf) (Food and Drug Administration Amendments Act of 2007 or FDAAA), Title VIII, Section 801 mandates that a "responsible party" (i.e., the sponsor or designated principal investigator) register and report results of certain "applicable clinical trials":   - **Trials of Drugs and Biologics:** Controlled, clinical investigations, other than Phase I investigations, of a product subject to FDA regulation; - **Trials of Devices:** Controlled trials with health outcomes of a product subject to FDA regulation (other than small feasibility studies) and pediatric post-market surveillance studies.   "Applicable clinical trials" generally include interventional studies (with one or more arms) of drugs, biological products, or devices that are subject to FDA regulation, meaning that the trial has one or more sites in the U.S, involves a drug, biologic, or device that is manufactured in the US (or its territories), or is conducted under an investigational new drug application (IND). | |

| Principal Investigator’s Assurance |
| --- |
| As the Principal Investigator I have ultimate responsibility for the performance of this study, the protection of the rights and welfare of the human subjects, and strict adherence by all co-investigators and research personnel to all IRB requirements, and all applicable federal and state regulations and laws for human subject research. I hereby assure the following:  - All named individuals on this project have read and understand the procedures outlined in the protocol. - All experiments and procedures involving human subjects will be done under my supervision or that of another qualified professional listed on this protocol. - No changes will be made to the approved protocol or consent form without prior IRB approval (except in an emergency to safeguard the well-being of subjects). - Only the most current, approved, IRB stamped consent form will be used to obtain informed consent from subjects or their legally authorized representative (unless waived by the IRB). - I will promptly report to the IRB in writing any protocol violations/deviations, unanticipated problems involving risks to subjects or others, and adverse events (AEs), within the time specified by IRB policy. - If I will be unavailable to direct this research personally, as when on leave or vacation, I will arrange for a co-investigator to assume direct responsibility in my absence. If this is not a co-investigator named in my absence, I will notify the IRB in writing of the responsible party. - I will obtain continuing review before the date approval for this study expires. I understand if I fail to apply for continuing review, approval for the study will automatically expire, and study activity must cease until current IRB approval is obtained.   6/17/15  Principal Investigator’s Signature Date |
|  |

| Faculty Sponsor’s Assurance (if applicable) |
| --- |
| By my signature, I certify that the student/investigator listed on page one is knowledgeable about the regulations and policies governing research with human subjects and has sufficient training and experience to conduct this particular study in accord with the approved protocol. In addition,   - I agree to meet with the student/investigator on a regular basis to monitor study progress. - Should problems arise in the course of the study, I agree to be available to personally supervise the student in solving them. - I assure that the student/investigator will promptly report to the IRB any protocol violations/deviations, unanticipated problems involving risks to subjects or others, and adverse events within the time specified by IRB policy. - If I will be unavailable, as on vacation, I will arrange an alternate faculty sponsor to assume responsibility during my absence and I will advise the IRB of such arrangements.     Signature of Faculty Sponsor Date  (if co-investigator is a student, resident, or fellow) |

| Statement of Financial Interests |
| --- |
| By the signatures below, each investigator is certifying that either no financial interest exists or a complete listing of all financial interests related to the proposed project is provided. All individuals named below further acknowledge their responsibility to disclose any new reportable financial interest obtained during the term of the project. The Principal Investigator’s signature also certifies that all individuals required to make disclosures have been listed below: (Attach additional sheet if necessary) |
| Do you, your spouse, or dependent children, have a financial interest in the work to be conducted under the proposed project?  No Yes: Attach Financial Disclosure Form  Signature of Principal Investigator Date  No Yes: Attach Financial Disclosure Form  Signature of Co-Investigator Date  No Yes: Attach Financial Disclosure Form  Signature of Co-Investigator Date  No Yes: Attach Financial Disclosure Form  Signature of Co-Investigator Date  No Yes: Attach Financial Disclosure Form  Signature of Co-Investigator Date  No Yes: Attach Financial Disclosure Form  Signature of Co-Investigator Date |

| Principal Investigator's Statement of Regulatory Compliance |
| --- |
| By my signature, I certify that:Neither I nor any other investigators on this study have been suspended or debarred under the Federal Food, Drug, and Cosmetic Act, 21 USC § 335a (a) or (b) or disqualified under 21 CFR § 312.70 or § 812.119.There are no pending regulatory for cause audits, investigations or proceedings involving study investigators relating to compliance with laws regarding the conduct of any clinical research. |
| Principal Investigator’s Signature Date |

| Documentation of Investigator Education in Human Subject Research |
| --- |
| Training in Human Research Subject Protections is required for all individuals who are participating in research activities at Children’s Hospital & Research Center Oakland and Children’s Hospital Oakland Research Institute. The Principal Investigator, Co-Investigators and other study staff interacting with research subjects must complete the University of Miami School of Medicine CITI Program in the Protection of Human Research Subjects to obtain IRB approval of a new study. Core Modules (Basic Course) must be completed only once, and Continuing Education Modules (Refresher Course) must be completed annually. (Attach additional sheet if necessary for other study personnel.) |
| Nooshin Razani, MD MPH X Basic/Refresher CITI Course Completed  Name of Principal Investigator       Kelley Meade, MD X Basic/Refresher CITI Course Completed  Name of Co-Investigator or Study Staff      Christine Schudel, Study Coordinator X Basic/Refresher CITI Course Completed  Name of Co-Investigator or Study Staff       Mindy Benson, Project Manager X Basic/Refresher CITI Course Completed  Name of Co-Investigator or Study Staff  Amaka Agodi, CHORI Intern X Basic/Refresher CITI Course Completed  Name of Co-Investigator or Study Staff  Maoya Alqassari, Research Assistant X Basic/Refresher CITI Course Completed  Name of Co-Investigator or Study Staff |
|  |

| Departmental Reviews |
| --- |
| **The following individuals must review your completed application, and initial this page,**  **before the approved study documents can be released by the IRB** |
| **IRB #:  2015-042     Principal Investigator:      Nooshin Razani, MD MPH**  **Study Title:      Stay Healthy In Nature Everyday (SHINE)**  ***Return Initialed Form to (study coordinator):       Ext.***  ********************************************************************************************************************  ***CHORI STAFF***  *(NOTE: IRB staff will forward the application to the CHORI staff (1- 4) after an initial review)*  1. Sponsored Programs Office, Associate Director (Cheng/Rosales)  2. Technology Transfer, Director -- Suzanne Haendel, JD N/A  *(Cheng/Rosales to check N/A if not required)*  3. V.P. Research Operations -- Rajnesh K. Prasad, MBA  4. Interim Executive Director and S.V.P., Research – Janet C. King, PhD  ********************************************************************************************************************  **HOSPITAL STAFF**  *(NOTE: PI and/or Study Coordinator must circulate a hard copy of the application, consent and protocol to the following departments (5 – 9) for their review, if required.*  5. Director, CHRCO Pharmacy Services or designated Research Pharmacist N/A  (if pharmacy resources will be used)  6. Medical Director, Pulmonary Center N/A  (if Respiratory Care Services or Pulmonary Function Lab will be used)  7. Medical Director, Cardiology (EKGs, Echos) N/A  8. Other CHRCO resources, (as applicable), e.g., clinical laboratory,  diagnostic imaging (**print name and department below**) N/A  UCSF Benioff Children’s Hospital Oakland Primary Care Clinic    9. Administrative Director CRC, Laurie Schumacher, PhD, MPH N/A  (if utilizing CTSI-PCRC resources) |

| **Summary Information** | | | | | | | | | | |  | |
| --- | --- | --- | --- | --- | --- | --- | --- | --- | --- | --- | --- | --- |
| **Age Range of Eligible Subjects:**  Subjects will include clinic patients and their guardians.  Clinic patients age 4 and older are eligible.  Patient guardians 18 and over are eligible. | | | | | | | | | | |  | |
| **Subject Population:** (Please check all that apply) | | | | | | | | | | |  | |
|  |  | | a. neonates | | X | f. minors | | |  | k. cancer patients | |  |
|  | X | | b. minorities/immigrants | |  | g. non-English speaking | | |  | l. terminally ill | |  |
|  |  | | c. normal volunteers | |  | h. students | | |  | m. wards of the court | |  |
|  |  | | d. institutionalized | |  | i. prisoners or parolees | | |  | n. pregnant women | |  |
|  |  | | e. decisionally impaired | |  | j. other | | | | | |  |
|  | **Study Type:**  If the research involves any of the following, please check all that apply. | | | | | | | | | | |  |
|  | |  | | a. Investigator-sponsored IND or IDE. A Sponsor-Investigator is an individual who both initiates and conducts a clinical trial. Regardless of funding source, the individual has the responsibilities of both a sponsor and investigator. | | | | | | | | |
|  | |  | | b. Investigational Drug (IND) | | |  | m. Investigational Device (IDE – HUD) | | | | |
|  | |  | | c. Genetic Research (DNA) | | |  | n. Vaccine Trial | | | | |
|  | |  | | d. Collection of Biological Specimens for Banking | | |  | o. Respiratory Treatments, Sleep  Studies, PFTs for clinical purposes | | | | |
|  | |  | | e. Collection of PHI (identified data) for Database | | |  | p. Gene Transfer Therapy | | | | |
|  | |  | | f. Collection of Remnant Surgical Specimens | | |  | q. Biohazardous Waste | | | | |
|  | |  | | g. Magnetic Resonance Imaging (MRI) | | |  | r. Radiation (including X-ray, DXA) | | | | |
|  | |  | | h. HIV Screening | | |  | s. HIV/AIDS Research | | | | |
|  | |  | | i. Alcohol and Drug Abuse Research | | |  | t. Controlled Substances | | | | |
|  | |  | | j. Acute Care Waiver of Informed Consent | | |  | u. Transplantation | | | | |
|  | |  | | k. Behavioral Observations | | |  | v. Deception | | | | |
|  | | X | | l. Surveys, Questionnaires or Psychological Testing | | |  | w. Audio/Videotapes or Focus Groups | | | | |

| **Data Safety Monitoring: *All interventional studies involving greater than minimal risk must include a Data Safety Monitoring Plan (DSMP).*** A DSMP is a plan established to assure that each research study has a system for appropriate oversight and monitoring of the conduct of the study to ensure the safety of participants and the validity and integrity of the data. The DSMP should indicate specifically whether there will be a formal Data Safety Monitoring Board (DSMB) or Data Monitoring Committee (DMC).  **All Investigator-sponsored IND or IDE studies must have a DSMP, including an SOP for data monitoring.** | | | | | | | | | |  |
| --- | --- | --- | --- | --- | --- | --- | --- | --- | --- | --- |
| Has a Data Safety Monitoring Plan been established to review data and/or adverse events related to this study? | | | | | | | X N/A (minimal risk)  Yes No | | |  |
|  | Describe the DSMP below: | | | | | | | | |  |
|  |  | | | | | | | | |  |
|  | **Research Sites:** Except for multi-center clinical trials (e.g., industry, COG) list all sites in which the research is to be conducted and attach other IRB approval letters. If applicable, attach letters of support from those institutions. X N/A – Multi-center clinical trial | | | | | | | | |  |
|  |  | | | | | | | | |  |
|  | **Investigational drugs/devices:** If any investigational drugs or biologic agents are used in this study, please include two copies of the Investigational Drug Information or Investigator’s Brochure (IB) with this application.  **X N/A IB Number:      Version Date of IB:**  **Complete and attach the following IRB forms (on the website) as applicable:**  **Study Review - Investigational Drug Information**  **Study Review - Investigational Device Information** | | | | | | | | |  |
| Protocol Summary | | | | | | | | | |  |
| **Protocol Version Date:       Amendment #:**  **Protocol Number:       N/A (no number)** | | | | | | | | | |  |
| Please complete the requested information in the categories below. If the item does not apply to your research, please indicate that the question is not applicable. The information should be intelligible to IRB reviewers from a variety of lay and scientific backgrounds. | | | | | | | | | |  |
| **Hypothesis:** Briefly explain the hypothesis(es) to be tested. If the study is not designed to test a hypothesis, simply state “None.” | | | | | | | | | |  |
| The SHINE study is a randomized controlled study of the effect of a park-based family support group on multiple outcomes (including stress and physical activity) in a low income population.   1. Primary hypothesis:   Children and caregivers enrolled in the SHINE program will have improved stress compared to those who are not enrolled in the SHINE program.   1. Secondary hypotheses:   Children and caregivers enrolled in the SHINE program will have improved social support, family functioning, physical activity, park visitation, and nature affinity compared to caregivers not enrolled in the program.  Children with obesity, ADHD, and asthma who participate in the SHINE program will show improvements in their symptoms.  The effect of the SHINE intervention will vary depending on the baseline life stressors present in the caregiver or child (the effects of nature interact with baseline stress and are dose dependent) and the dose of nature received.  During a SHINE outing, participants will experience immediate improvements in stress and 30 minutes of moderate-to-vigorous physical activity. | | | | | | | | | |  |
| **Purpose of the study:** What are the specific scientific aims of this study? | | | | | | | | | |  |
| Aim1: To determine changes in stress, social support, family function, physical activity, park visitation, and nature affinity amongst primary care patients ages 4 and older, and their caregivers enrolled in the SHINE intervention compared to those in a control group.Aim 2: To determine changes in BMI, ADHD symptoms, and asthma control for patients enrolled in the SHINE program compared to those who are not.Aim 3: To assess immediate changes in stress and minutes spent in moderate-to-vigorous physical activity by enrolled patients and their enrolled parents during SHINE outings. | | | | | | | | | |  |
| **Background and Significance:** Include a brief summary of previous work that provides a basis for the proposed research and that supports the expectations of obtaining useful information without undue risk to human subjects. **Provide a Bibliography (References)**  This information aids IRB reviewers in assessing how valuable the project is likely to be. If graphs or tables are used to convey information, please maintain a consistent style and make sure that fonts are no less than 11-point in size. If no preliminary data are available, it may be helpful to indicate briefly why this proposed study is a reasonable starting point. Note that some IRB members are non-scientists and may not be familiar with scientific or technical terms. | | | | | | | | | |  |
| Families served by UBCHO Primary Care Clinic experience high levels of stress (Children’s Hospital Community Needs Assessment, 2013). As a safety-net clinic, UBCHO PCC serves a diverse set of patients, with a common thread of poverty (Children’s Hospital Community Needs Assessment, 2013). Without intervention, stress can lead to a variety of physical and mental health morbidities (Brotman, Golden and Wittstein, 2007) (Bovier, Chamot and Perneger, 2004) (Marin, et al. 2011).  Local parks have the potential to serve as an affordable, community-based resource in stress management for low-income communities in that they can provide families with an opportunity to be with friends and family in nature.  Nature has been shown to buffer the effects of stress on health. Like other protective factors, such as a caring adult, safe play places, greater stimulation, the presence of greenspace has been empirically linked to greater resilience (Bradely et al. 1994). Nancy M. Wells and Gary W. Evans showed in 2003 that nature acts as a buffer or moderator, mitigating the impact of stress on adversity on children - in other words the presence of a natural environment attenuated the adverse effects of strayers or other adverse main effects on health or well-being. In a study of 337 children in 3-5th grade, they found that socio-economic status was positively related to children’s self-worth (F 15.86, p<0.001), there was a main effect of nature such that the higher nature corresponded to higher self worth.  Factors that contribute to nature’s buffering effect on stress include social cohesion and support, physical activity, and improved attention when groups are in nature.  Increased social cohesion has been shown to be a mediator in the overall pathway linking exposure to nature to an increase in health benefits (Sjerp de Vries, et al. 2013). The tie linking green spaces and improved social cohesion is well documented. In one study, 83% more social activities were found to occur in green spaces than in barren ones with twice as many people using the green versus barren spaces (Sullivan, Kuo and al 2004). In another, green spaces were shown to be more supportive of children’s play and gave them more intergenerational interaction with adults, including non-family members (Faber Taylor and Wiley 1998). These and other studies suggest that green spaces serve as social magnets, bringing people together in a natural environment where social interaction and community building can organically occur.  Research to date suggest that participation in outdoor activities facilitates a sense of interconnectedness with nature (Scott, Amel and Manning 2014). This sense of attachment, we postulate, may also provide a form of social support.  Time outdoors in natural spaces is associated with increases in physical activity. One study, for example, found that for every additional hour spent outdoors, physical activity increased by 27 minutes a week and the prevalence of overweight decreased by 41% to 27% (Cleland, et al. 2008). Physical activity, in turn, lesses anxiety and depression, lower rates of obesity, and improved cardiovascular functioning (US Department of Health and Human Services 2008). | | | | | | | | | |  |
| **Study Design:** (Check all that apply). | | | | | | | | | |  |
| Placebo | | | Blinded | X Randomized | Investigational intervention without random assignment | | | | |  |
| If this study has any of the formal designations below, please indicate below: N/A | | | | | | | | | |  |
| Phase I | | | Phase II | Phase III | Phase IV | Open Label Extension | | | |  |
| Additional description of general study design. Sequentially list all procedures, drugs or devices to be used on human subjects. Describe any use of placebos and indicate whether subjects will be randomized in this study. Attach flow diagram if appropriate. **If there are any investigational drugs, devices or biologic agents used in this study, complete and attach the FDA Form 1572.** If this is an investigator-initiated study, attach the FDA Investigational Drug Application (FDA Form 1571). | | | | | | | | | |  |
| To evaluate specific aims 1 and 2, the study design uses a randomized controlled trial design.  Potential participants will be given a short description of the study (Please see attached waiver to screen patients). If they agree to be screened, they will be screened for eligibility using a short screening tool.  If they are eligible based on the screening tool, they will consented and enrolled. All enrolled participants (which includes a caregiver and a child per family), will receive:   - Resources to find local parks - A recommendation to be physically active three times a week - A pedometer with a diary to record their daily steps - Three tubes for salivary cortisol collections   They will be asked to:   - Complete a questionnaire - Collect a first morning salivary cortisol during the first week, fourth week, and twelfth week of participation - Return to clinic to complete a questionnaire, and to have their child’s BMI measured, on the fourth week and twelfth week of participation - Turn in their physical activity logs on the fourth and twelfth week visits   Upon completing the baseline measures, participants will be randomized to one of two treatment groups ('A' or 'B') by a computer program (we will use Redcap) and the result recorded. Randomization will be blocked (using random permuted blocks) to ensure that the groups are balanced periodically.  The control group will have no added activities.  The intervention group will be asked to return to clinic on the first, second, and third week of the study at 1 pm - 4pm on Saturday. During these visits, they will meet with clinic staff as well as with other participating families. They will turn in their physical activity logs for a week. They will then board a school bus and travel to one of three local parks (Week 1 = Crab Cove, Week 2 = Temescal Lake, Week 3= Redwood Regional Park). Once at park, they will experience:   1. A warm welcome and ice breaker games 2. 30 minutes of lunch and unstructured play 3. 60 minutes of light walking   They will journal about their outdoor experiences. The journal will include questions about stress and well-being.  The intervention group will also be encouraged to visit a park or be physically active at least three times a week every week for six weeksand will be able to chronicle their outdoor physical activity in their journal.  To assess Aim 3 we will survey only the families in the intervention arm:  During the third SHINE outings, enrolled guardians and children will be invited to wear an accelerometer for the duration of the event. Accelerometers will be programmed to record data in 15-second intervals and will allow us to calculate minutes spent in moderate to vigorous physical activity. Participants will return their accelerometers prior to boarding the shuttles to return home. | | | | | | | | | |  |
| Study Population | | | | | | | | | |  |
| **Characteristics of the Subject Population:**  Describe the gender, ethnic background and health status. Provide a candid discussion of potential problems, if any, related to the study population. Explain the rationale for the use of special classes such as pregnant women, children, prisoners, wards of the court, or other vulnerable populations. If women, minorities, non-English speaking subjects or children are excluded, provide written justification. | | | | | | | | | |  |
| The target population for this study are low income children and caregivers. The study population are UCSF Benioff Children’s Hospital Oakland Primary Care (UBCHO PCC) patients ages 4 and older, and a caregiver. The study population approximates the target population as 95% of patients live within 300% of the federal poverty level. We will be looking at children above age three in order to assess physical activity. Our study population excludes physically disabled patients because we are following physical activity as an outcome.  UBCHO PCC patients are very diverse, with African American and Latino patients comprising more than half of the patient population. UBCHO patients are linguistically diverse, with 58 reported languages; one in five patients speaks a language other than English. Spanish is the second most common reported language after English. For this reason, we will be translating all SHINE materials into Spanish, and we will have Spanish speaking park personnel available during all outings at the parks. Arabic speaking patients are also increasing in numbers at UBCHO Primary Care Clinic. As our research assistant Maoya Alqassari is a fluent Arabic speaker, she will be of assistance in ensuring that this population is not excluded. | | | | | | | | | |  |
| **Inclusion/Exclusion Criteria:** Indicate the criteria for exclusion and inclusion and explain the system for equitable selection of subjects. | | | | | | | | | |  |
| Our system for equitable selection of study patients is to use a screening tool that will ensure systematic enactment of our inclusion and exclusion criteria.Inclusion criteria are: the adult must be the legal guardian, at least 18 years old and familiar with the child’s health. The enrolled child must be at least 4 years old. This age cut-off for participating children was chosen because of programming considerations once the families are in the parks and in order to measure physical activity in the child. Because we are measuring physical activity, the caregiver and child need to have no physical disabilities that would limit their ability to be able to be physically active.  Both caregiver and child must be willing to be randomized, and must be available for follow up over the next 3 months. If they are randomized to the intervention group must return to clinic weeks 1, 2, 3 on Saturdays for group park visits, as well as to visit parks on their own for six weeks. | | | | | | | | | |  |
| 1. **Eligibility for Study:** How is eligibility determined and by whom? | | | | | | | | | |  |
| Eligibility for the study will be determined by the research coordinator by reading through the eligibility screen with referred patients and their guardians. All answers to the eligibility questions must be yes. If any of them are no, the patient will not be eligible. Any questions will be discussed with the study coordinator Christine Schudel or by the PI, Dr. Razani. | | | | | | | | | |  |
| 1. **Duration of Subjects' Participation in the Study (include follow-up if applicable):** | | | | | | | | | |  |
| Patients will be enrolled for a total of 12 weeks. They will participate in a survey at the time of randomization, at one month out from the beginning of the study, and three months after the end of the intervention. | | | | | | | | | |  |
| Enrollment Plan and Recruitment | | | | | | | | | |  |
| 1. **Planned Enrollment at CHRCO:**  Number of subjects needed to complete the study. | | | | | | | | 200 total participants (100 families with 1 adult and 1 child enrolled in each family) | |  |
| **Enrollment plan:** If you expect failed screenings or subject withdrawals, will they be replaced until the appropriate numbers of subjects have completed the study? If No, explain below. | | | | | | Yes XNo N/A | | | |  |
| We need 200 participants (100 parent/child dyads) in order to complete this goal. In order to enroll this number, we anticipate screening 300 participants (150 parent/child dyads) for eligibility, enrolling 240 participants (120 parent/child dyads) and having 200 participants (100 parent/child dyads) participate in the programing.  If a subject fails a screening, we will keep record of their screening, and will continue screening until appropriate subjects have enrolled in the study.  Because our intervention arm has park outings that occur at a set time (either in July, August, or September), we may not be able to replace all subject withdrawals. We will have the option of increasing the number of participants in later sessions if the first session is under-enrolled. | | | | | | | | | |  |
| **Recruitment:** What methods will be used to identify and recruit potential subjects? Attach a copy of all planned advertisements, flyers and letters, etc. to potential subjects. | | | | | | | | | |  |
| Recruitment will occur through provider referral and self-referral. In order to advertise the study:We will present at staff meeting for health care providers. Potential participants referred by health care providers will be called by the research assistant.The research assistant will be present in the clinic waiting room during the first two weeks of each month.Dr. Razani, the research coordinator or research assistant will approach patients who are waiting for their appointment, will describe the project, and ask their interest in being involved.Patients will be allowed to self-refer. A recruitment flier will be placed in the waiting room with the study coordinator’s phone number listed  - Patients will be given the opportunity to consent right there and then, or will have the opportunity to go home, consider, and call back later for screening/consent. | | | | | | | | | |  |
|  | Informed Consent | | | | | | | | |  |
|  | 1. **Capacity to Consent:** Will all subjects have the capacity to give informed consent? If not, describe the likely range of impairment and explain how, and by whom, their capacity to consent will be determined. | | | | | | | | |  |
|  | Potential participants will be screened for eligibility prior to consent. We have submitted a request to waive screening prior to screening for eligibility. A short description of the study will be given prior to completing the consent form. This way, we will minimize the time burden placed on potentially interested families. In addition, the eligibility criteria limits participating caregivers to those who are also the child’s legal guardian. These individuals are also in charge of the child’s medical decision making and will be able to consent for the child. It will be clear that the legal guardian is expected to accompany the child on park outings and on follow up visits.  If they are eligible and interested, all subjects will have the capacity to give informed consent in English, Spanish, or Arabic. If they require assistance in another language, an interpreter will be called. After a summary of the protocol is given, the participant will be asked by the study personnel to summarize their understanding of the study, and whether they have any questions.  The adult participant will be giving consent for themselves and for their child. Children above 13 will also sign the informed consent form. Children ages 7 - 13 will have the study described to them (they will be read an assent form, submitted with this application), and will give a verbal assent.  The subject’s capacity to give consent will be determined by the person administering the eligibility screen and consent discussion. This will be our research assistant, our research coordinator, or the principal investigator. Each of these individuals is trained and experienced in interview techniques. Any questions or grey areas will be discussed with the principal investigator. | | | | | | | | |  |
|  | 1. **Translation of Consent Form:** If you anticipate that more than five (5) subjects in the study population will speak/read a single language other than English, the IRB-approved English language consent form must be translated according to IRB policy. Please indicate below into what language(s) the consent form will be translated, and the method of translation you are requesting. | | | | | | | | |  |
|  | N/A - Unanticipated at this time. Translation will be submitted later if required.  **Language(s):**      Spanish  X Certified Translator, accompanied by an "Affidavit of Accuracy". **Required for IND/IDE study**; may be used for any study.  Qualified Translator, according to the IRB SOP "Translation of Study Documents". This process may be used for all studies that do not involve an IDE or IND. | | | | | | | | |  |
|  | 1. **Study Personnel Administering the Consent Process:** Please identify by name and credentials the individual(s) who will be authorized to describe the research to subjects or their representatives, and to invite their participation. To insure that subjects give complete informed consent and are able to ask and have answered all questions regarding the nature of their participation, the personnel administering the consent must have appropriate training and background. | | | | | | | | |  |
|  | Nooshin Razani, MD MPH. Dr. Razani has served as PI of several studies and has received training in informed consent. These include a study of high risk behaviors amongst injection drug users in Tehran, Iran.  Study Coordinator: Christine Schudel, MSW, MPH is the Program Director of the Family Information & Navigation Desk (FIND) at UCSF Benioff Children's Hospital Oakland. Christine is also the Education Specialist for the Primary Care Clinic (PCC) at CHO. As the Education Specialist, she oversees health education programs at PCC and has been in this role since earning her Masters in Social Welfare and Public Health in 2012 from the University of California, Berkeley. Christine has served as research coordinator for numerous studies.  Research Assistant: Maoya Alqassari is currently working in the Primary Care Clinic as an Arabic Translator for the International Clinic, and helps families administer developmental questionnaires in Arabic. She will be trained by Dr. Razani and by Christine Schedel in questionnaire administration. During her experiences in the primary care clinic she has come across a variety of clinical scenarios and is aware of protocols for sensitive questions. | | | | | | | | |  |
|  | 1. **Process of Consent:** Please discuss how the consent process will be conducted, describing the following elements: 2. The environment and location where the informed consent will be solicited; 3. Opportunities for the potential subjects to discuss their participation with family or others before signing the consent form; 4. How and by whom it will be determined whether the subject or their legally authorized representatives understand the information provided; and 5. **The types of forms used** (e.g., adult consent form, parental permission form, combined form, information sheet with waiver of documentation of consent) | | | | | | | | |  |
|  | Potential participants will be informed about the study in these settings:  In the clinic waiting room while awaiting their medical appointments  In the clinic room while meeting with their health care provider  By telephone. If a health care provider recommends they participate in the study, we will contact them by phone.  We have submitted for a request to waive consent prior to screening for eligibility. If this is granted, we will give the potential participants a short description of the study (the script of this description is included in the eligibility criteria). If they are interested in being screened for eligibility, we will complete a short eligibility screen. If they are interested and eligible to participate, they will be invited to complete the consent form.  We will complete the consent process in person, in a quiet and private space. If the initial discussion is in the clinic, they will be invited to a separate study area to complete the consent. If the initial discussion is by telephone, we will make an appointment for them to come into clinic for consent and enrollment.  The eligibility criteria limits participating caregivers to those who are also the child’s legal guardian. The caregiver will be asked whether they are the legal guardian. This information will then be checked at the clinic registration desk. As these individuals are also in charge of the child’s medical decision making, they will be able to consent for the child. Of note, it will be made clear that the legal guardian is expected to accompany the child on park outings and on follow up visits.  If they are eligible and interested, all subjects will have the capacity to give informed consent in English, Spanish, or Arabic when our interpreter is present. If they require assistance in another language, an interpreter will be called. After a summary of the protocol is given, the participant will be asked by the study personnel to summarize their understanding of the study, and whether they have any questions.  The adult participant will be giving consent for themselves and for their child. Children above 13 will also sign the informed consent form. Children ages 7 - 13 will have the study described to them (they will be read an assent form, submitted with this application), and will give a verbal assent. | | | | | | | | |  |
|  | 1. **Assent of Minor:**  For subjects age 7 through 17: N/A (under 7 or adult) | | | | | | | | |  |
|  | | a) Considering the subject’s potential capacity and medical condition, what is the suggested age of the minors to provide assent? | | | | | | | |  |
|  | | b) Detail below whether the assent should be in writing (a separate assent form signed by the child), and/or obtained orally. | | | | | | | | |
|  | | Assent will be obtained for children ages 7-17 through a written form. | | | | | | | | |
|  | | c) Would it would be appropriate to include on the consent form a signature block for adolescents who are able to understand the adult consent form **(minimal risk studies only).** | | | | | | | X Yes  No | |
|  | d) Detail any justification for requesting that the IRB waive assent. N/A | | | | | | | | |  |
|  |  | | | | | | | | |  |
|  | 1. **Information Withheld from Subjects:**  If any information about the research purpose and design of the study will be withheld from subjects, please explain the non-disclosure and describe plans for post-study de-briefing. N/A | | | | | | | | |  |
|  | Non-disclosure will include randomization.  If the participant agrees, post-study debriefing will include making a report available to the participant’s health care provider regarding their change in BMI and physical activity. | | | | | | | | |  |
| Risk and Benefit Assessment | | | | | | | | | |  |
| 1. **Potential Risks and Discomforts:** Describe any potential risks or likely adverse effects of the drugs, biologics, devices or procedures subjects may encounter in the study. State the potential risks – physical, psychological, social, legal or other – connected with the proposed procedures and assess their likelihood and seriousness. | | | | | | | | | |  |
| - Inconvenience, other activities they may miss - Answering sensitive questions about their health and well-being - While they are at the park, they may experience physical discomfort, may be at risk for sunburn, or may have asthma or allergy flares. Less likely is that they may get hurt. | | | | | | | | | |  |
|  | 1. **Safety Precautions for Minimizing Risks:** Describe the procedures for minimizing any potential risks. Where appropriate, discuss provisions for ensuring necessary medical or professional intervention in the event of adverse effects to the subject. | | | | | | | | |  |
|  | 1. We will minimize inconvenience to the families by allowing them to bring other family members to the park outings and park visits. We will also provide a meal and snacks at park outings and clinic visits. 2. To ensure confidentiality of patients, no names will be collected. Instead, a unique code will be created for each guardian, with a linkable unique code for each child. 3. Our team will be trained to ask questions in a confidential and nonjudgmental way in private 4. We will be clear that our study discusses stress, family function, and potentially sensitive topics while obtaining informed consent. We will alert families to the fact that their answers are confidential, but since we are mandated reporters, we will need to break confidentiality in the case of harm to a minor. A clear reporting protocol is in place and attached. 5. We will give adequate information about what will happen at the parks, how to be prepared, what clothing, snacks, and medications to bring when you go outdoors. Specifically, we will instruct participants to bring sunscreen, hats, their allergy and asthma medications with them to park outings. 6. In the case that a participant has forgotten sunscreen, we will some available during the park outings. | | | | | | | | |  |
|  | 1. **Benefit Ratio:** What is the risk benefit ratio of this research, compared with available alternatives? Describe the potential benefits the subjects may receive as a result of their participation in the research and what benefits to society may be expected. **For greater than minimal risk research involving children there must be the prospect of direct benefit to the individual subjects.**   Note: The potential benefits of the research must justify the risks to human subjects. The risk benefit ratio of the research must be at least as favorable for the subjects as that presented by standard treatments for their condition. When comparing the risk/benefit ratio of research with that of available alternatives, the alternative of doing nothing should be included in the analysis. | | | | | | | | |  |
|  | The benefits of this study include:   - The potential to learn about parks and local natural resources. - Receiving advice and support in becoming more physically active. - The potential to develop friendships and social support with other study participants. - Scientific discovery of the health benefits of nature potentially facilitating funding of future programs to benefit similar families. | | | | | | | | |  |
|  | 1. **Therapeutic Alternatives:** What therapeutic alternative(s) are reasonably available to potential subjects should they choose not to participate in the study? These may be research or non-research based alternatives. | | | | | | | | |  |
|  | Caregivers or children who are noted to report high levels of loneliness, stress, or family dysfunction will be referred to existing services through the behavioral health department at UCSF Benioff Children’s Hospital Oakland. If any acute psychosocial issues are noted, they will be referred to the social worker on call. Patients in the control arm will have access to the Family Information and Navigation Desk as well as to East Bay Regional Park District resources if they seek assistance in arranging for outdoor physical activity experiences. | | | | | | | | |  |
|  | **Financial Considerations** | | | | | | | | |  |
|  | 1. **Payment for Participation:** Describe all plans to compensate subjects, including provision of services, and other reimbursements. Describe the conditions that subjects must fulfill to receive full or partial pro-rated payment. | | | | | | | | |  |
|  | - Participating families will receive $20 for completing the baseline survey, $40 for completing the second survey, and $40 for completing the final survey. This will result in $100 compensation for their time during the duration of the study. Please note that this incentive is per family, and not per participant. - Each participating caregiver will receive a pedometer. - Each participating child will receive a pedometer. - Families in the intervention arm will receive a lunch during the park outings. | | | | | | | | |  |
|  | 1. **Financial Obligations of Subjects:** Will subjects have to pay for any of the tests or treatments that they receive as part of the research? Please clarify who will pay for the procedures associated with the study as well as procedures that may be part of standard clinical care. Clarify that insurance and other third party payers may not cover standard procedures if they are associated with a research project. | | | | | | | | |  |
|  | Participating families do not have to pay to participate. | | | | | | | | |  |
|  | 1. **Emergency Care and Compensation for Research-Related Injury:** If the research presents an unknown or greater than minimal risk of illness/injury, the financial liability for the costs of care associated with the potential research related illness/injury must be specified. Industry sponsors are required to cover the cost of treating any injury caused by study drug or procedures. For non-industry studies if no funds are set aside, please include template language from the consent form. | | | | | | | | |  |
|  | This research presents minimal risk of illness/injury. | | | | | | | | |  |
| Research Methods and Procedures | | | | | | | | | |  |
| 1. **Methodology and Data Collection:** Describe the research procedures that will be followed. Please list, in sequence, all study procedures, tests, and treatments required for the study. Please indicate those that are experimental and those that may be considered standard treatment. Include a detailed explanation of any experimental procedures. Attach table if available. Describe all activities involving human subjects and explain the frequency and duration of each activity. | | | | | | | | | |  |
| Table 1 presents a timeline of when data will be collected. The experimental group and control group will follow the same timeline.  1. A questionnaire will be administered at baseline, four weeks, and twelve weeks. The survey questions are detailed in the following question. The survey administers will be blinded to randomized assignment.  2. A saliva sample will be obtained at baseline, four weeks, and twelve weeks. Patients will be given a 1.5 ml tube and asked to gather early morning saliva. This sample will be gathered by the research staff on the day of their follow up appointments, who will then store the sample in a freezer at CHORI until it is sent for cortisol level.  3. All participants will be given a pedometer and a daily log of their steps. They will self-record the number of steps they take each day.  4. During the third SHINE outings, participating caregivers and patients will be invited to wear an accelerometer for the duration of the event. Accelerometers will be programmed to record data in 15-second intervals and will allow us to calculate minutes spent in moderate to vigorous physical activity. Participants will return their accelerometers prior to boarding the shuttles to return home. Families will also receive a journal, in which they will be asked to record their stress before and after the outing.  5. Weight and height will be completed using the measuring tools available at the Primary Care Clinic. | | | | | | | | | |  |
| 1. **Surveys, questionnaires, or psychological tests:**  If applicable, please describe the provisions for administering these measures, the mode of administration, the setting, and if special training or qualifications are necessary. N/A | | | | | | | | | |  |
| Surveys will be administered in-person and at clinic. The adult questionnaire will take 20-30 minutes of time. The child questionnaire will take 10 minutes, and can be administered simultaneously to the adult questionnaire. We will use tablets to administer the questionnaire, with paper versions available to those who would prefer to fill the survey themselves on a paper version.  Special training or qualifications are not necessary to administer these tests. However, as several of the questions include sensitive topics, a clear protocol is in place for dealing with potential issues that may come up. The population serves by the UCSF Benioff Children’s Hospital Oakland experiences high levels of stress related to poverty. For that reason, we anticipate that a significant portion of respondents will have high stress scores. We will also be asking about social isolation and loneliness. We anticipate that a proportion of respondents will answer that they feel socially isolated. We will also be asking about childhood adversity, which includes questions about domestic violence, community violence, and abuse in the home. We anticipate that the proportion answering that these adverse events have happened to the enrolled child will mirror, at least, national averages of 8% of respondents for each of these questions (Child and Adolescent Health Measurement Initiative, 2013, available at; [www.childhealthdata.org](http://www.childhealthdata.org)).  In order to properly support families who disclose sensitive issues, we will enact a very clear protocol. For families with high stress, social isolation, or potentially reportable conditions, the principal investigator will always be contacted. The principal investigator will screen the family for safety. The principal investigator Dr. Razani, is currently an attending pediatrician in the adolescent medicine and homeless clinics. She has experience enacting safety screens and mandatory reporting protocols through her experiences in these clinics.  All families who reveal stress, loneliness, or adversity, will be given mental health resources. If the patients additionally screen positive for a reportable condition, or if there is concern for their current safety, the principal investigator will contact the on-call social worker. The social worker at the Primary Care Clinic will then work with the principal investigator to enact mandatory reporting. If the social worker is not available, the principal investigator will contact the hospital’s social work office to find back up social work support.This protocol is the same protocol used by all mandatory reporters, including clinicians in the primary care clinic.  Physical Activity will be measured through self-report of pedometer readings. The parent will be responsible for recording the number of steps walked daily in their own journal. The caregiver will be responsible to helping children enter their number of steps in the child’s own journal. If the child is old enough to enter their own steps, that is acceptable. Both the caregiver and the child will be trained to use the pedometer and the journal upon enrollment in the study.  Salivary cortisol will be measured through obtaining early morning saliva samples. Caregivers and children will be expected to submit saliva samples. They will be trained to do so by the research assistant during study enrollment, and will have a phone number to call in case they have any questions.  Physical activity will also be measured through accelerometry. Research staff will place and remove accelerometers for enrolled caregivers and children during one of the park outings. | | | | | | | | | |  |
| 1. **Data Storage:** Please complete the following questions regarding data storage: | | | | | | | | | |  |
| a. How will the data be collected and recorded? How will the data be coded to protect personal privacy? | | | | | | | | | |  |
| All data will be recorded on REDCap, an electronic data capture system that is HIPAA compliant and allows for output of efficient data management and quality checks in a cloud-based environment. For situations where REDCap is not available, or if the participant requests, the questionnaires will be administered by paper, and then transferred to REDCap by a trained research coordinator.  Participants will be assigned a unique identifying code. Caregivers and children will have a linked code, in order to follow them as a pair throughout the study. The key linking the code to the patient’s name, and linking the patient’s name to their enrolled caregiver’s name, will be kept on a secure file in the Children’s Hospital shared hard drive. | | | | | | | | | |  |
| b. How will the data be stored during the study? | | | | | | | | | |  |
| Data will be stored on RedCap, as well as on an encrypted and password protected shared drive on the Children’s Hospital hard drive. | | | | | | | | | |  |
| c. Who will have access to the data and the data codes? If data with subject identifiers will be released, specify the person(s) and agencies to whom this information will be released. | | | | | | | | | |  |
| The Principal Investigator, Nooshin Razani, the research Coordinator Christine Schudel, the Research Assistant Maoya Alqassari and a statistician will have access to the data. Only the principal investigator and research coordinator will have access to the code linking patient name to patient code. | | | | | | | | | |  |
| d. What will happen to the data when the study is completed? | | | | | | | | | |  |
| When the study is complete, the data will be stored in an encrypted and password protected CHO server for 5 years and then destroyed/deleted completely. | | | | | | | | | |  |
|  | | | | | | | | | |  |

| Data Analysis |
| --- |
| 1. **Statistical Analysis:** Please delineate the data analysis plans for this study. Include planned statistical analyses and explanation of determination of sample size. Briefly describe what statistical analysis(es) of which outcome will be applied to address each primary aim. Examples of statistical analyses include:   *Calculation of descriptive statistics such as mean, median, SD, range, tallies.*  Examination of graphs such as outcome vs. time, scatterplots of two variables, Kaplan-Meier curves.  Estimation of differences between two groups with comparison by t-test or Mann-Whitney test.  Estimation and testing of within-person changes by matched t-test or Wilcoxon signed-rank test.  Multiple linear regression, logistic regression, or Cox proportional hazards regression.  Repeated measures models (usually requires the help of a statistician).  For qualitative research, briefly describe how qualitative data will be analyzed. |
| We will examine differences in outcome variables between the guardians participating in the SHINE intervention and the control group and will stratify by age, If caregiver stress improves for participants in SHINE intervention decreases more than guardians in the control arm, we infer a functional relationship between the SHINE intervention and guardian stress.  We will present descriptive statistics of baseline covariates using means and proportions.  Our primary outcome (guardian stress) is measured by the PSS score, a continuous repeated measure. Because this study uses a randomized control design, we will use ANOVA procedures to estimate the effect of SHINE. We will also adjust for possible empirical confounding by incorporating covariates with a bivariable association with the primary outcome (guardian stress) into a multivariable ANOVA model.  Analyses will also explore potentional interaction that correspond to amount of pre-existing life stressors or ACE score, and nature exposure and the effect of SHINE on stress. We will estimate the degree to which measures of pre-existing stress account for the effects attributed to the particular SHINE.  .  Secondary outcomes of interest (child stress, BMI, ADHD score, asthma score) are also continuous measurements and will be analyzed using ANOVA procedures or other appropriate multivariable models accounting for correlations due to repeated measurements. We will also adjust for possible empirical confounding by incorporating covariates with a bivariable association with the secondary outcomes.  Aim 3. We will examine changes in stress, and minutes spent in moderate to vigorous physical activity duringhe SHINE intervention for participating guardians and children.  We will present changes in these continuous variables using a paired-t test.  Power Analysis  We calculated the power to detect differences of 0.5 effect size between the intervention and control group. With a type I error of 0.05, and a Type II error rate of 0.20, and a two-tailed statistical test.  Attrition and Missing Data  We expect 20% attrition among participants, and we do not expect participant attrition to vary by treatment condition - those receiving the intervention may have higher attrition than others. Both groups have the inconvenience of coming into clinic. One group has the extra time to spend, but it is also a bigger commitment. In light of attrition, however, we will attempt to identify key predictors of attrition status, such as [example predictors, e.g., SES, reading level], and test for differences between conditions. Our plan to deal with missing data is to analyze patients as intent to treat and will check for the impact of missing data using sensitivity analysis. |

| Comments/Remarks |
| --- |
| This area may be used as continuation of other items. You may also attach additional sheets as necessary. |
|  |

| Attachments |
| --- |
| Please list [Attachments, Supplements and Appendices](http://www.research.ucsf.edu/chr/forms/chrnewappatt.asp), including Version(s) and date(s). |
| Table 1. Outcome variable, measurement tool, and timing  Figure 1. Parent predictor and outcome variables  Figure 2. Child predictor and outcome variables  Attachments:   1. SHINE Postcard_061815 2. SHINE Activity Journal_062515 3. Recruitment Flier_062515   4. Caregiver Survey_062515  5. Child Survey_062515  6. SHINE protocol for mandated reporting_06282015 |
|  |

| Study Abstract (For CHORI Website) |
| --- |
| Provide an abstract/full synopsis of this study to be posted on the CHORI Website. Examples are at:  http://www.chori.org/Clinical_Studies/Active_Studies/active_studies_home.html |
| Despite mounting evidence that nature matters for human health, we are not aware of any prospective intervention trials looking at the use of nature as a health intervention. Like other protective factors, such as the presence of a caring adult, safe play places, greater stimulation, the presence of green-space in a child’s life has been empirically linked to greater resilience. Nature has been proposed as a buffer to stress through several pathways: by providing a space for friends and families to gather (thereby increasing social support and improving family relationships), by increasing opportunities for physical activity, and by improving cognition. Research to date suggests that participation in outdoor activities facilitates a sense of connectedness to place. This sense of attachment, we postulate, may also provide a form of social support. Families served by Children’s Hospital Oakland Primary Care Clinic experience high levels of stress. As a safety-net clinic, the primary care clinic serves a diverse set of patients, with a common thread of poverty. The Stay Healthy In Nature program was developed to encourage stress management skills through play and physical activity in parks. It was formed through a collaboration between health care providers at Children’s Hospital Oakland Primary Care Clinic and the staff at East Bay Regional Parks District (EBRPD). This six week program uses a combination of facilitated and independent outings into local parks to encourage sustained behavior change and measurable health benefits. The SHINE study is a randomized controlled study of the effect of this park-based family support group on multiple outcomes (including stress and physical activity) in a low income population. The study is supporting by EBRPD and the National Regional Parks and Association, and will take place during summer and fall 2015. |

| Table 1. Outcome variable, measurement tool, and timing | | | |
| --- | --- | --- | --- |
| Outcome variable | measurement tool | short description | timing |
| Parental Stress | PSS-10 (Lee, 2012) | 10 item scale used to measure one's self-report of stress. Reliability and validity scores have been performed for adults | Baseline, 1 month, 3 months |
| Child Stress | PSQ 8-11 | 19 item self-report. 9 items measure psychological stress, while 10 items measure physiological stress | Baseline, 1 month, 3 months |
| Parental and child stress | Salivary cortisol | Participants will bring in early am cortisol | Baseline, 1 month, 3 months |
| Family Cohesion | FACES IV (Olson, 2011) | 7 item balanced cohesion scale taken by parents | Baseline, 1 month, 3 months |
| Connection to Nature | (Adults) Love and Care for Nature (Perkins, 2010) | 15 item self-report scale used to measure emotional connection to nature, tested for people 18 and older | Baseline, 1 month, 3 months |
|  | (Children) Connection to Nature Index (Bragg et al., 2013) | 16 item scale developed and tested for children aged 8-12 | Baseline, 1 month, 3 months |
| Physical Activity | Pedometry | Journals for use at home | Daily record for 6 weeks by participants in intervention arm |
|  | Accelerometry | Armbands placed during park outings | Week 3 outing |
| Increased Park Usage | One week recall self-report |  | Baseline, 1 month, 3 months |
| Obesity | BMI | Take their weight and height | Baseline, 1 month, 3 months |

Figure 1. Parent predictor and outcome variables


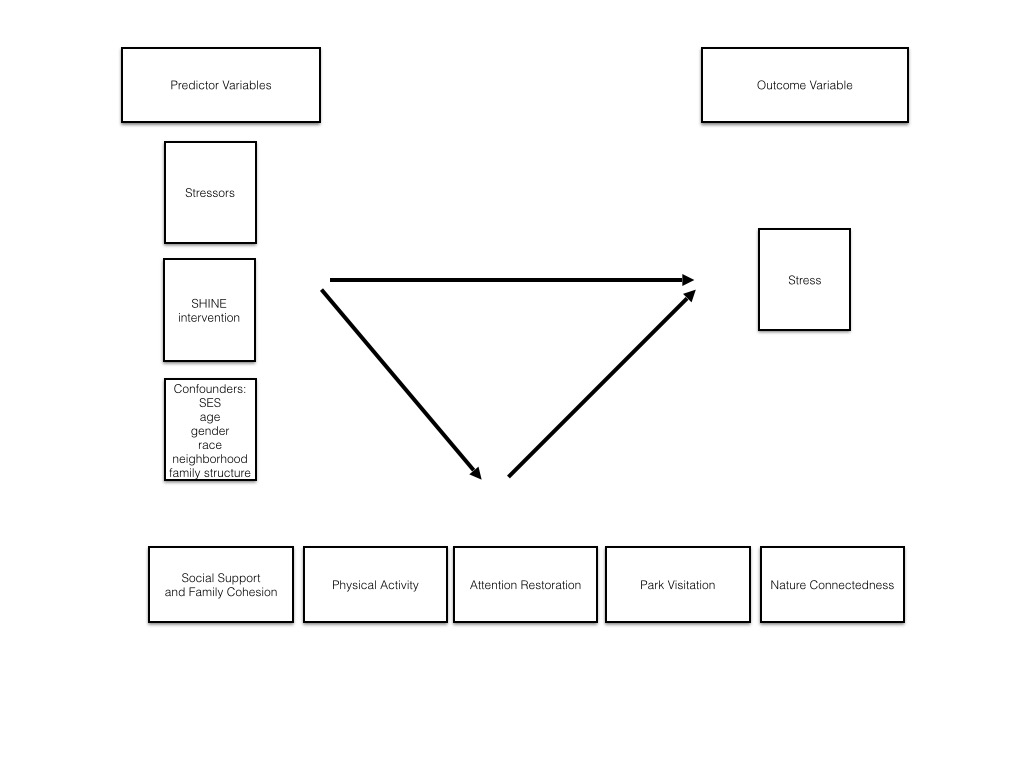


Figure 2. Child predictor and outcome variables


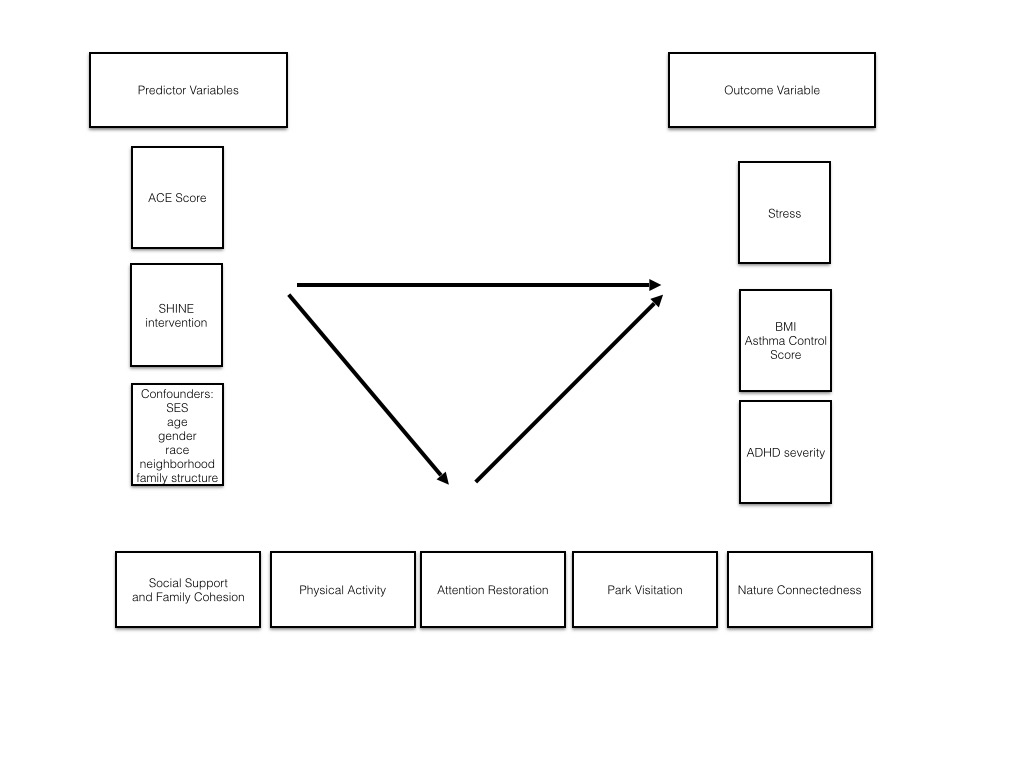

Supplement: S3 File — (DOCX) [file pone.0192921.s003.docx]
